# Supplementary material for: Integrin β3 directly inhibits the Gα13-p115RhoGEF interaction to regulate G protein signaling and platelet exocytosis
Source: Nat Commun. 2023 Aug 16;14:4966. doi: 10.1038/s41467-023-40531-3 (PMC10432399; doi:10.1038/s41467-023-40531-3)
Supplement: Supplementary file 1 — Supplementary Information [file 41467_2023_40531_MOESM1_ESM.pdf]

Integrin β_3 directly inhibits the $G\alpha_{13}$-p115RhoGEF interaction to regulate G protein signaling and platelet exocytosis.

Yaping Zhang[1#], Xiaojuan Zhao[1#], Bo Shen[1#], Yanyan Bai[1], Claire Chang[1], Aleksandra Stojanovic[1,2], Can Wang[1], Andrew Mack[1], Gary Deng[3], Randal A. Skidgel[2], Ni Cheng[1] and Xiaoping Du[1,*]

[1]Department of Pharmacology and Regenerative Medicine, University of Illinois at Chicago, Chicago, IL 60612, USA

[2]Dupage Medical Technology, Inc., Chicago, IL 60612, USA

[3]Eli Lilly, Indianapolis, IN, 46285, USA

[#]Equal contribution authors.

[*]Correspondence to Xiaoping Du: xdu@uic.edu (X.D)

This file includes Supplementary Figures 1-6.

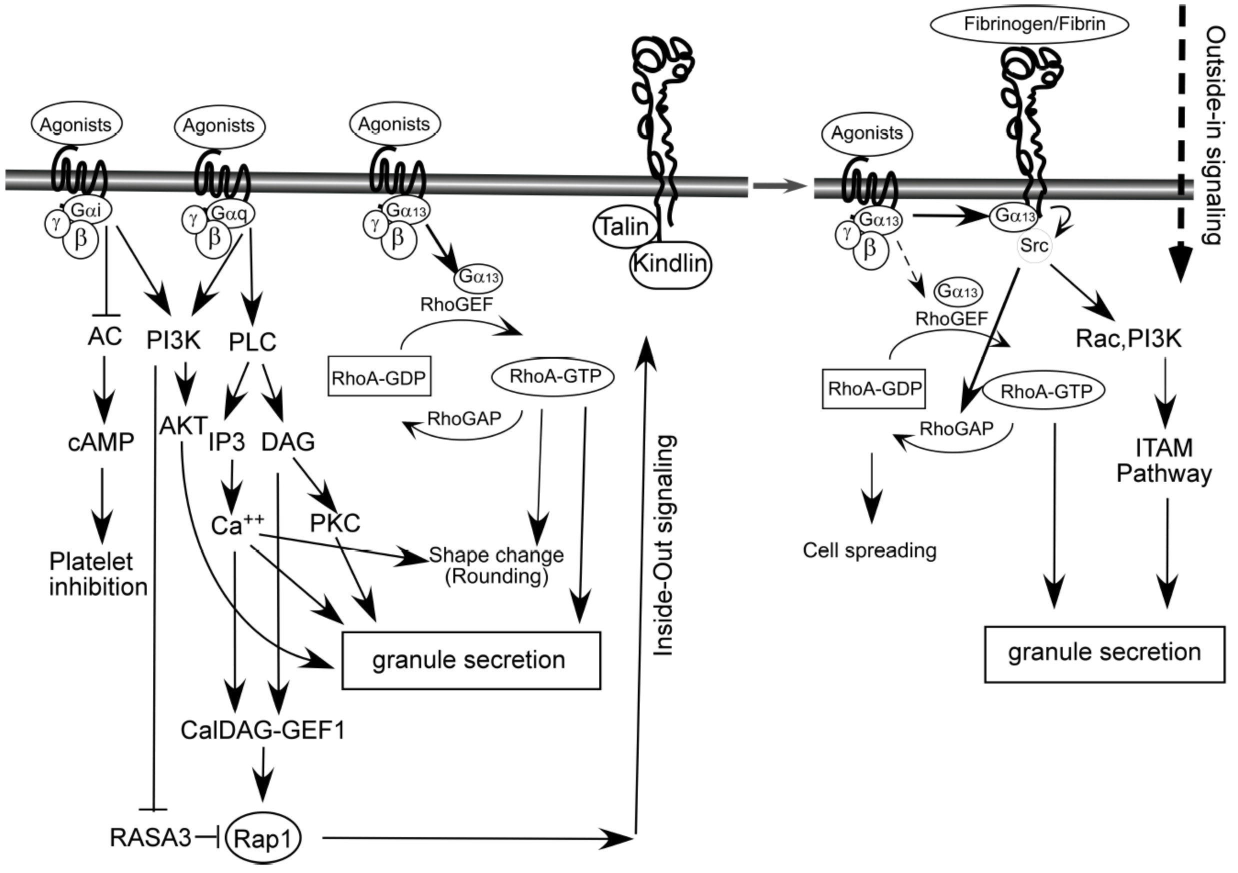

Supplemental Fig. 1 Integrin "inside-out" and "outside-in" signaling. Several platelet agonists, including thrombin, activate platelets by binding to the G-protein-coupled receptors (GPCRs). GPCRs, through the Gα$_{13}$-RhoGEF-RhoA pathway, stimulate contractility and integrin-independent granule secretion. GPCRs, via Gαi and Gαq, facilitate Rap1 activation, which induces talin and kindlin binding to the cytoplasmic domain of β$_3$, resulting in integrin activation. This process is referred to as integrin inside-out signaling. Following integrin ligation, Gα$_{13}$ binds to the integrin β$_3$ cytoplasmic domain, which mediates integrin outside-in signaling by activating Src, which subsequently activates p190RhoGAP, leading to RhoA inhibition and induction of cell spreading. Src also activates ITAM signaling pathway via Rac, PI3K and reactive oxygen species, leading to integrin-dependent granule secretion, greatly amplifying platelet aggregation and thrombus formation. Many other molecules are also involved in integrin signaling, although their roles in the integrin-G protein crosstalk are unclear or they are known constituents of the described pathways. For clarity, these molecules are not labeled in the schematic.

a

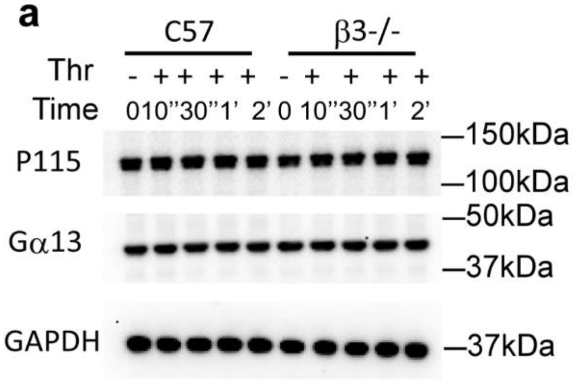

Supplemental Fig. 2 p115RhoGEF and RhoA expression in WT and $\beta3^{-/-}$ platelets. a Washed WT and $\beta_3^{-/-}$ platelets were stimulated with 0.02U/mL of thrombin for the indicated time points. Total platelet lysates were electrophoresed and probed with anti-p115 RhoGEF, anti-Gα_{13}, or anti-GAPDH by Western blotting. The blots are run on different gels using the same samples as Fig. 1d. The experiment was performed once to verify similar levels of GAPDH in different samples as an additional processing control. Source data are provided as a Source Data file.

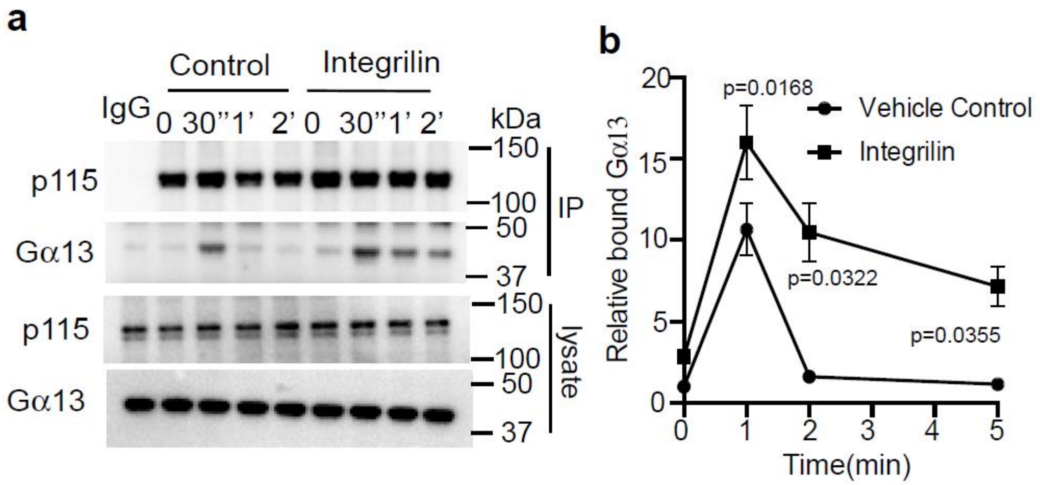

Supplemental Fig. 3 Eptifibatide (integrilin) enhances thrombin- induced Gα_{13} binding to p115-RhoGEF in mouse platelets.

a,b Co-immunoprecipitation of p115RhoGEF and Gα_{13} in mouse platelets pre-treated with Integrilin (40 µg/ mL) or vehicle control (CA, 5.25 mg%) stimulated with thrombin (0.02 U/ mL) for the indicated time points. Platelets were pretreated with 500 µM aspirin to exclude the differential influence of secondary TXA_2 production on the Gα_{13} pathway. **a** a representative Western blot; **b** quantification of data from 3 independent experiments. Data are shown as mean ± SEM. Data were analyzed using Student's *t*-test (paired), two tailed. Source data are provided as a Source Data file.

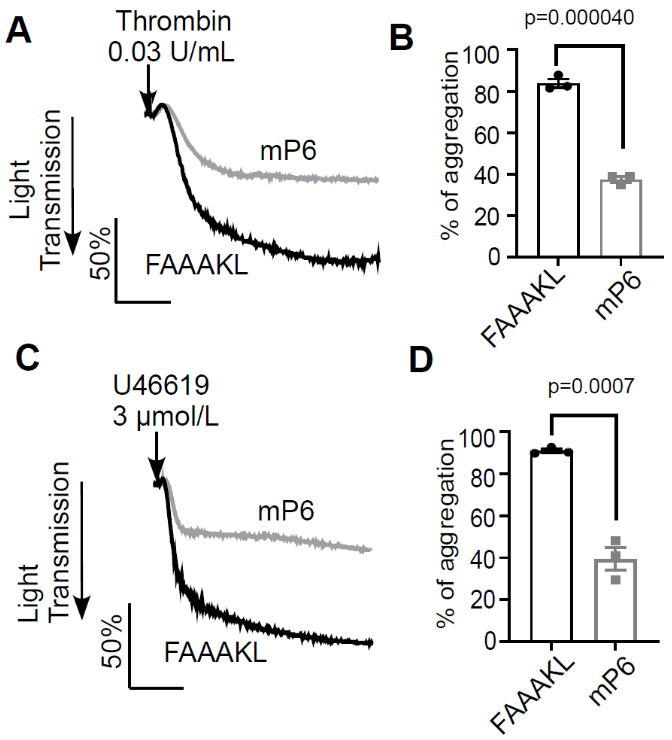

Supplemental Fig. 4 The effects of β_3 ExE motif peptide mP6 on platelet aggregation in washed platelets in Tyrode's solution with fibrinogen added (100 µg/ml). a,**b** real time traces (**a**) and quantification (**b**) of aggregation of platelets preincubated with 20 µM control FAAAKL or mP6 HLPNs and stimulated with 0.03 U/ mL thrombin. **c**,**d** real time traces (**c**) and quantification (**d**) of aggregation of platelets preincubated with 20 µM control FAAAKL or mP6 HLPN and stimulated with 3 µM U46619. **b**,**d** n=3, independent experiments. All data are shown as mean ± SEM. Statistical significance was determined using Student's *t*-test, two tailed. Note that primary platelet aggregation occurs in the presence of mP6. Source data are provided as a Source Data file.

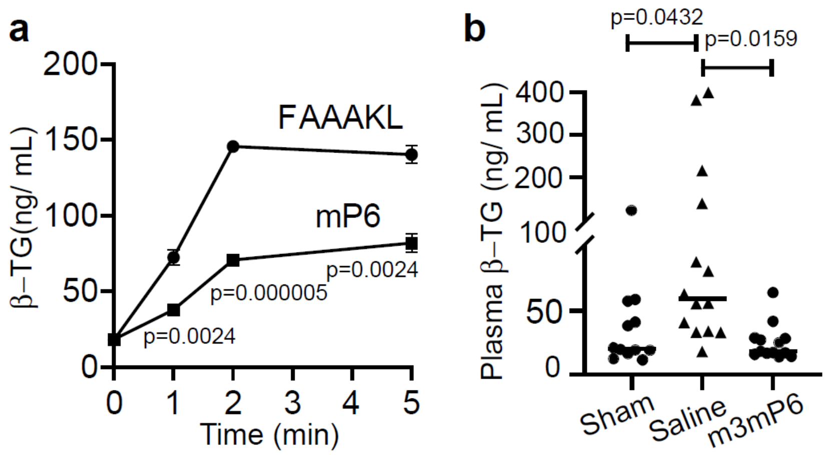

Supplemental Fig. 5 Gα_{13} antagonistic peptide mP6, inhibits platelet α granule secretion of β-TG *in vitro* and *in vivo*. a, effects of control FAAAKL or mP6 HLPNs (20 μM) on β-TG secretion, in mouse platelets stimulated with 0.03U/ mL thrombin in an aggregometer stirring at 37°C and 1000 rpm. n = 3, independent experiments. Data are shown as mean ± SEM**. b**, plasma β-TG levels in sham, saline and M3mP6 HLPN treatment groups 24 hours after myocardial infarction with reperfusion. Sham group, n=12; Saline group, n=14; M3mP6 group, n=13, individual animals. Data are shown as median ± SEM. Data were analyzed using Student's *t*-test, two tailed. Source data are provided as a Source Data file.

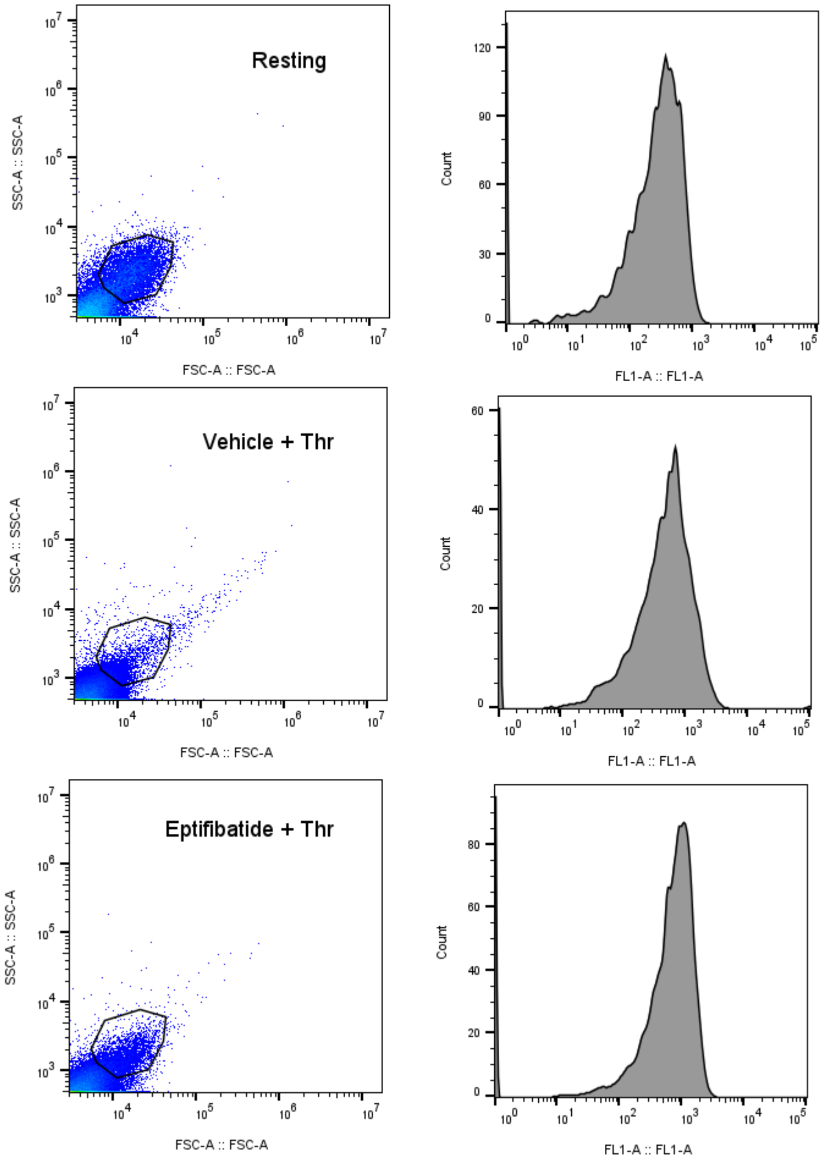

Supplemental Fig. 6 Gating used for flow cytometry analysis. Gating strategy used for surface P-selectin expression on mouse platelets induced by thrombin (0.03U/ mL). Washed platelets were pretreated with vehicle control or Eptifibatide (20 μg/ mL) for 3 minutes, and then stimulated with thrombin in an aggregometer at 37°C at 1000 rpm stirring rate for 10 minutes.
