# Supplementary material for: Integrin β3 directly inhibits the Gα13-p115RhoGEF interaction to regulate G protein signaling and platelet exocytosis
Source: Nat Commun. 2023 Aug 16;14:4966. doi: 10.1038/s41467-023-40531-3 (PMC10432399; doi:10.1038/s41467-023-40531-3)
Supplement: Supplementary file 3 — Reporting Summary [file 41467_2023_40531_MOESM3_ESM.pdf]

nature portfolio

Corresponding author(s): Xiaoping Du
Last updated by author(s): Jul 18, 2023

Reporting Summary

Nature Portfolio wishes to improve the reproducibility of the work that we publish. This form provides structure for consistency and transparency in reporting. For further information on Nature Portfolio policies, see our Editorial Policies and the Editorial Policy Checklist.

Statistics

For all statistical analyses, confirm that the following items are present in the figure legend, table legend, main text, or Methods section.

n/a	Confirmed	
☐	☒	The exact sample size (*n*) for each experimental group/condition, given as a discrete number and unit of measurement
☐	☒	A statement on whether measurements were taken from distinct samples or whether the same sample was measured repeatedly
☐	☒	The statistical test(s) used AND whether they are one- or two-sided<br>*Only common tests should be described solely by name; describe more complex techniques in the Methods section.*
☒	☐	A description of all covariates tested
☐	☒	A description of any assumptions or corrections, such as tests of normality and adjustment for multiple comparisons
☐	☒	A full description of the statistical parameters including central tendency (e.g. means) or other basic estimates (e.g. regression coefficient) AND variation (e.g. standard deviation) or associated estimates of uncertainty (e.g. confidence intervals)
☐	☒	For null hypothesis testing, the test statistic (e.g. *F*, *t*, *r*) with confidence intervals, effect sizes, degrees of freedom and *P* value noted<br>*Give P values as exact values whenever suitable.*
☒	☐	For Bayesian analysis, information on the choice of priors and Markov chain Monte Carlo settings
☒	☐	For hierarchical and complex designs, identification of the appropriate level for tests and full reporting of outcomes
☒	☐	Estimates of effect sizes (e.g. Cohen's *d*, Pearson's *r*), indicating how they were calculated

Our web collection on statistics for biologists contains articles on many of the points above.

Software and code

Policy information about availability of computer code

Data collection	Detailed in Methods section. For In vitro binding and ELISA assays, FlexStation3 Multi-mode Microplate Reader from Molecular Devices, LLC. was used; For platelet aggregation and secretion assay, Aggro/Link8 for Windows (version 1.2.9) from CHRONO-LOG CORP. was used; For flow cyotmetry analysis, CFlow Plus software (version 1.0.227.4) from BD Biosciences was used.
Data analysis	For Western blot image analysis, ImageJ software (version 1.53e) from NIH was used. For FACS data, FlowJo 10.9.0 Software was used. For data statistic analysis, GarphPad Prism (version 9) from GraphPad Software, G*power 3.1.9.7 software from HHU, Excel (office 365) from Microsoft, Inc. were used.

For manuscripts utilizing custom algorithms or software that are central to the research but not yet described in published literature, software must be made available to editors and reviewers. We strongly encourage code deposition in a community repository (e.g. GitHub). See the Nature Portfolio guidelines for submitting code & software for further information.

Data

Policy information about availability of data

All manuscripts must include a data availability statement. This statement should provide the following information, where applicable:

- Accession codes, unique identifiers, or web links for publicly available datasets
- A description of any restrictions on data availability
- For clinical datasets or third party data, please ensure that the statement adheres to our policy

The data that support the findings in the current study are available in the Article and its Supplementary Information files. Additional information can be obtained

from the corresponding author upon reasonable request.

Research involving human participants, their data, or biological material

Policy information about studies with human participants or human data. See also policy information about sex, gender (identity/presentation), and sexual orientation and race, ethnicity and racism.

Reporting on sex and gender	Adults of either sex who are in good health
Reporting on race, ethnicity, or other socially relevant groupings	Adults of any race and any ethnic category who are in good health
Population characteristics	healthy adults (21-64 years old) who are not using any medication. Ideal blood donor should have<br>· No hepatitis, HIV exposure<br>· No bleeding conditions<br>· No high/low blood pressure history<br>· No diabetes<br>· No blood transfusion in past 12 month<br>· No dental procedures and oral surgery in past 7 days<br>· No immunization in past 56 days<br>· No anti-platelet drug treatment in the past 2 weeks.
Recruitment	The healthy donors were randomly recruited without any self-selection by the performer. Blood drawings were performed by a certified phlebotomist.
Ethics oversight	Human Subjects Research Protocol was approved by IRB of Office for the Protection of Research Subjects at University of Illinois at Chicago.

Note that full information on the approval of the study protocol must also be provided in the manuscript.

Field-specific reporting

Please select the one below that is the best fit for your research. If you are not sure, read the appropriate sections before making your selection.

☒ Life sciences ☐ Behavioural & social sciences ☐ Ecological, evolutionary & environmental sciences

For a reference copy of the document with all sections, see nature.com/documents/nr-reporting-summary-flat.pdf

Life sciences study design

All studies must disclose on these points even when the disclosure is negative.

Sample size	Sample sizes were determined according to previous studies published by our group (Cheng et al. Nat Commun.2021,12:3185 and Pang et al. 2020, Sci. Transl. Med.12,eaaz7287) or others. For in vitro studies, all the experiments were independently repeated at least three times to obtain data for statistical analyses (student t test, two paired). For experiments using mouse platelets, platelets were isolated and pooled from 3-6 mice each group and treated as one independent experiment. Mice were assigned into different groups on the basis of genotypes and randomized within given age with equal amount of genders. For experiments using human platelets, platelets were isolated from 1 donor per time and treated as one independent experiment. For in vivo MI/R experiment, C57BL/6J mice were assigned into different treatment groups on the basis of randomized within given age with equal amount of genders. Per animal protection requirement and when scientifically valid, data collected in identical in vivo experiments are used for multiple purposes to avoid unnecessary mouse sacrifice. Statistical significance between two groups was analyzed using student's t test, two tailed. The sample size were predicted with preliminary data by using G*power 3.1.9.7 software, t-tests (Means: difference between two independent means, two groups) , when given tails(two), effect size d (calculated from each group mean and SD), alpha error prob (0.05), power (0.8) and N number of each group. When the actual sample size > predicted sample size and the actual power > 0.8, it suggests the sample sizes are sufficient.
Data exclusions	No data were excluded.
Replication	The majority data in the study were repeated at least three times to ensure the reproducibility. Proper statistical analyses were applied to illustrate significance when it's needed. Some Key experiments were repeated by different persons in the group and all the attempts were successful.
Randomization	All animals were grouped on the basis of genotypes within given age with equal amount of genders. All mice in the different treatment groups were also randomly assigned with similar age and equal amount sexes. For experiments using mouse platelets, mouse platelets were isolated and pooled from 3-6 mice and treated as one independent experiment. The samples of human platelets were obtained by isolating from one donor at a time. In order to ensure unbiased allocation, different groups were assigned to utilize the same platelets within each experiment. Donors participated in experiment randomly.
Blinding	The MI/RI treatment was processed blinded to surgery performer. In vitro experiments were not blinded because blinding is not effective in improving objectivity in these experiments and inefficient.

Reporting for specific materials, systems and methods

We require information from authors about some types of materials, experimental systems and methods used in many studies. Here, indicate whether each material, system or method listed is relevant to your study. If you are not sure if a list item applies to your research, read the appropriate section before selecting a response.

Materials & experimental systems

n/a	Involved in the study	
☐	☒	Antibodies
☒	☐	Eukaryotic cell lines
☒	☐	Palaeontology and archaeology
☐	☒	Animals and other organisms
☒	☐	Clinical data
☒	☐	Dual use research of concern
☒	☐	Plants

Methods

n/a	Involved in the study	
☒	☐	ChIP-seq
☐	☒	Flow cytometry
☒	☐	MRI-based neuroimaging

Antibodies

Antibodies used

1. Mouse anti-Ga13 (67188-1-lg, clone 2G8F10), Rabbit anti-p115 RhoGEF (11363-1-AP), Mouse anti-GAPDH (60004-1-lg, clone 1E6D9) and Rabbit anti-integrin β3 (18309-1-AP) were purchased from Proteintech (Rosemont, IL).
2. Rabbit IgG (2729S) and Rabbit anti-p115 RhoGEF (D25D2) were purchased from Cell Signaling technology Inc. (Danvers, MA).
3. FITC-conjugated rat anti-mouse CD62P antibody (553744,clone RB40.34) and FITC-conjugated rat IgG1 control (553995, clone A110-1) were purchased from BD Pharmingen.
4. Anti-RhoA mab(ARH05, clone 54D6.1.16) from RhoA activation assay Biochem Kit (BK036) was purchased from Cytoskeleton,Inc (Denver, CO).
5. Peroxidase IgG Fraction Monoclonal Mouse Anti-Rabbit IgG, light chain specific (code 211-032-171, lot 134305) and Peroxidase AffiniPure Goat Anti-Mouse IgG, light chain specific (code 115-035-174, lot 134893) were purchased from Jackson ImmunoResearch Inc.

Validation

1. Mouse anti-Ga13 (67188-1-lg) was used to blot mouse Ga13 in western blot assay (1:1000). Product information and citations can be found at: https://www.ptglab.com/products/GNA13-Antibody-67188-1-Ig.htm
2. Rabbit anti-p115 RhoGEF (11363-1-AP) was used to immunoprecititate p115RhoGEF from mouse platelets (2 micro gram / 500 micro liter cell lysate). Product information and citations can be found at : https://www.ptglab.com/products/ARHGEF1-Antibody-11363-1-AP.htm
3. Mouse anti-GAPDH (60004-1-lg) was used to blot mouse GAPDH in western blot assay (1:50000). Product information and citations can be found at : https://www.ptglab.com/products/GAPDH-Antibody-60004-1-Ig.htm
4. Rabbit anti-integrin β3 (18309-1-AP) was used to blot mouse integrinβ3 in western blot assay(1:1000). Product information and citation can be found at: https://www.ptglab.com/products/ITGB3-Antibody-18309-1-AP.htm
5. Rabbit IgG (2729S) was used for co-immunoprecipatation control (2 micro gram / 500 micro liter cell lysate). Product information and citation can be found at: https://www.ptglab.com/products/ARHGEF1-Antibody-11363-1-AP.htm
6. Rabbit anti-p115 RhoGEF(D25D2) was used to blot mouse p115RhoGEF in western blot assay(1:1000). Product information and citation can be found at: https://www.cellsignal.com/products/primary-antibodies/p115-rhogef-d25d2-xp-rabbit-mab/3669
7.FITC-conjugated rat anti-mouse CD62P antibody (553744) was used to label and detect platelet surface P-selectin expression(1 micro gram/ 10^5 platelets). The product information, applications and citations can be found at: https://www.bdbiosciences.com/en-us/products/reagents/flow-cytometry-reagents/research-reagents/single-color-antibodies-ruo/fitc-rat-anti-mouse-cd62p.553744
8.FITC-conjugated rat IgG1 control antibody (553995) was used in flow cytometry analysis to serve as a control for detecting P-selection expression(1 micro gram/ 10^5 platelets). The product information, applications and citations can be found at:https://www.bdbiosciences.com/en-ca/products/reagents/flow-cytometry-reagents/research-reagents/flow-cytometry-controls-and-lysates/fitc-rat-igg1-isotype-control.553995
9. Anti-RhoA mab(ARH05) was used to blot mouse RhoA in western blot assay(1:500). Product information and citations can be found at :https://www.cytoskeleton.com/arh05
10. Peroxidase IgG Fraction Monoclonal Mouse Anti-Rabbit IgG, light chain specific (code 211-032-171, lot 134305) was used as second antibody in western blot (1:3000). Product information and citations can be found at:https://www.jacksonimmuno.com/catalog/products/211-032-171.
11.Peroxidase AffiniPure Goat Anti-Mouse IgG, light chain specific (code 115-035-174, lot 134893) as used as second antibody in western blot (1:3000). Product information and citations can be found at:https://www.jacksonimmuno.com/catalog/products/115-035-174.

Animals and other research organisms

Policy information about studies involving animals; ARRIVE guidelines recommended for reporting animal research, and Sex and Gender in Research

Laboratory animals

Mouse colony C57BL/6J (stock # 000664), PF4cre (stock # 008535) and β3-/- mice (stock # 008819) were purchased from Jackson Laboratory. The Ga13 flox/flox mice were a gift from Dr. Stefan Offermanns' lab (Max Planck Institute for Heart and Lung Research,

	Bad Nauheim, Germany). All the mice of 8- to 16-weeks-old with an equal sex ratio were used in the studies and housed and bred in Biologic Resources Laboratory at University of Illinois at Chicago under 12 hours light dark cycles, control temperature (~23 degree) and 40-50% humidity with free access to food and water.
Wild animals	This study did not involve the use of wild animals.
Reporting on sex	Mice used in this study were with an equal sex ratio.
Field-collected samples	This study did not involve the use of field-collected samples.
Ethics oversight	Animal usage and protocol were approved by the Institutional Animal Care Committee, University of Illinois at Chicago. A randomized approach of choosing mice was used throughout the study, using all mice with the correct genotype without bias.

Note that full information on the approval of the study protocol must also be provided in the manuscript.

Flow Cytometry

Plots

Confirm that:

- [x] The axis labels state the marker and fluorochrome used (e.g. CD4-FITC).
- [x] The axis scales are clearly visible. Include numbers along axes only for bottom left plot of group (a 'group' is an analysis of identical markers).
- [x] All plots are contour plots with outliers or pseudocolor plots.
- [x] A numerical value for number of cells or percentage (with statistics) is provided.

Methodology

Sample preparation	Fresh mouse blood was drawn from the inferior vena cava and added with one-ninth volume of anti-coagulant solution (85 mM trisodium citrate, 83 mM D-glucose, and 21 mM citric acid). After adding a final concentration of 0.1 micro gram/mL prostaglandin E1 and 1 U/mL apyrase, platelet-rich plasma (PRP) was isolated by centrifugation of whole blood at 200g at 22° C for 10 minutes, and mouse platelets were further isolated by centrifugation at 870g at 22°C for 10 minutes. An additional 1 U/ mL apyrase and 1/100 volume of 0.5 M EDTA (pH 8.0) was added to PRP before centrifugation to minimize platelet activation. Thereafter, mouse platelets were washed once with CGS buffer (sodium chloride 0.12 M, D-glucose, 0.03 M, trisodium citrate 0.0129 M, pH 6.5) and resuspended in modified Tyrode's buffer (12 mM NaHCO3, 138 mM NaCl, 5.5 mM glucose, 2.9 mM KCl, 0.42 mM NaH2PO4, 10 mM N-2-hydroxyethylpiperazine-N'-2-ethanesulfonic acid, 1 mM MgCl2, pH 7.4). Washed platelets were then allowed to rest at room temperature for at least 1 hour. CaCl2 (1 mM) was added to platelet suspensions 10 minutes before use.<br>For P-slectin expression detection, washed platelets in Tyrode's buffer were pre-incubated with control vehicle or 20 micro gram/mL Eptifibatide, 20 micro mole control peptide or mP6 HPLNs for 3 minutes at room temperature, and then stimulated with thrombin in an aggregometer at 37°C at 1000 rpm stirring rate for the indicated time points. After fixing with 2% paraformaldehyde, platelets were incubated with an FITC-conjugated rat anti-mouse P-selectin antibody or rat IgG control for 30 min at 22°C in the dark. After dilution 10-fold in PBS (containing 1% BSA), platelet P-selectin expression was analyzed using a Accuri C6 flow cytometer with CFlow Plus software (version 1.0.227.4) (BD Biosciences).
Instrument	Accuri C6 flowcytometry
Software	CFlow Plus software (version 1.0.227.4) from BD Biosciences for collection and Flow Jo (Version 10.9.0) for analysis.
Cell population abundance	90~95% of live mouse platelets were validated by anti-CD62P staining.
Gating strategy	Platelets gating strategy was routinely used in our lab as published in various publications (Flevaris et al. Blood.2009, 113(4): 893-901 and Cheng et al. Nat Commun.2021,12:3185).The preliminary gating strategies are shown in Supplementary Fig. 6

- [x] Tick this box to confirm that a figure exemplifying the gating strategy is provided in the Supplementary Information.
